# Autogenous Cross-Linking of Recycled Keratin from Poultry-Feather Waste to Hydrogels for Plant-Growth Media

**DOI:** 10.3390/polym13203581

**Published:** 2021-10-17

**Authors:** Markus Brenner, Oliver Weichold

**Affiliations:** Institute of Building Materials Research, RWTH Aachen University, Schinkelstraße 3, D-52062 Aachen, Germany; brenner@ibac.rwth-aachen.de

**Keywords:** poultry feathers, waste utilisation, renewable materials, biobased materials, keratin hydrogel, keratin extraction, super-absorber, water absorption, plant growth, controllable swelling

## Abstract

The global rise in atmospheric temperature is leading to an increasing spread of semi-arid and arid regions and is accompanied by a deterioration of arable land. Polymers can help in a number of ways, but they must not be a burden to the environment. In this context, we present herein a method by which goose feathers, representative of keratin waste in general, can be transformed into hydrogels for use as a plant growth medium. The treatment of shredded feathers in Na_2_S solution at ambient conditions dissolves approx. 80% of the keratin within 30 min. During evaporation, the thiol groups of cysteine reoxidise to disulphide bridges. Additionally, the protein chains form β-sheets. Both act as cross-links that enables the formation of gels. The drying conditions were found to be crucial as slower evaporation affords gels with higher degrees of swelling at the cost of reduced gel yields. The cress germination test indicated the absence of toxic substances in the gel, which strongly adheres to the roots. Thereby, the plants are protected from drought stress as long as the gel still contains moisture.

## 1. Introduction

With an annual world production of more than 1 × 10^9^ t, keratin is one of the most abundant biopolymers [1]. The fibrous, water-insoluble material is found as a structural protein mainly in the animal kingdom [2], e.g., in the form of hair (including wool), feathers, claws, nails and horns [3]. In contrast to most other proteins, keratin contains high amounts of cysteine of up to 17% [4]. The thiol groups of cysteine are easily oxidised to disulphide bridges, which serve as intra- or intermolecular cross-links. This renders keratin materials water insoluble, chemically inert, tough, and strong [3]. The secondary structure is mainly governed by α-helices (e.g., in wool) or β-sheets (e.g. in feathers) as well as hydrogen bonding [5]. Due to their structure, keratins also show a noticeable resistance to common proteolytic enzymes, which causes keratin to only slowly degrade in nature [6]. Once the hydrogen bonds and disulphide bridges are broken, keratin behaves like a common denaturised protein in terms of solubility and digestibility by proteolytic enzymes [7]. Feathers make up for approximately 10% of poultry’s weight [8] and the estimated annual amount of feathers produced in poultry farms is approx. 5 million tons [9]. This is a common problem in poultry farms, as the huge amounts of feather waste is generally disposed of in landfills. Ways to deal with this increasing amount of keratin, thereby reducing the waste and at the same time generating economical welfare, are highly needed. There are some approaches to utilise shredded feathers as filler in petrochemical [10] and biobased thermoplastics [11]. Besides that, the addition of plasticisers, such as water and/or glycerol, allows ground feathers to be processed by standard thermoplastic equipment [12,13,14]. The sorption properties of feathers were successfully used to prevent the previously observed frothing of bio-based polycondensation-type thermosets and at the same time accelerate the polycondensation reaction [15]. Some research focuses on the application of feathers and feather meal as nitrogen fertilizer [16,17]. To control the nitrogen release, steam hydrolysis or crosslinking (e.g., by glutaraldehyde) were tested [18,19]. However, since the use of feathers in their natural, i.e., cross-linked form is an obstacle for most applications, they need to be solubilized [4]. As this is the key step to produce new keratin-based materials, it is the focus of many publications. Methods range from hydrothermal [20] or alkaline hydrolysis of the backbone [21], reductive cleavage of the disulphide bridges [22], sulfitolysis [23], enzymatic digestion [24], or solubilisation in ionic liquids [25]. However, the soluble proteins obtained with these methods differ in terms of amino acid composition and properties. A gentle method with only minimal changes to the primary sequence is the reductive cleavage. Here, in contrast to other methods, the cystine cross-links are broken reversibly and can reform upon oxidation. This provides the means to assemble stable materials from the dissolved protein [26]. The Shindai method [27] is one of the most widely used methods for keratin extractions and does not damage the protein backbone in the extraction process. For this, 2-mercaptoethanol or dithioerythritol are used as mild reducing agents in combination with highly concentrated urea solutions and a surfactant to facilitate the extraction. The extraction is followed by dialysis to yield a highly pure keratin solution. The reductive extraction can also be performed with sodium sulfide as shown by Poole and Church [28,29]. By substituting 2-mercaptoethanol by sodium sulfide, they were able to achieve high amounts of extracted keratin with less molar equivalents of reducing agent and at the same time to accelerate the extraction. The obtained keratin was used to produce high strength films as potential substitutes for commercial plastics [29]. Dissolved keratin is also used for the preparation of films [28], fibers [30], scaffold materials [31], hydrogels [32] and bioabsorbent materials [33]. During the production of wood-fibre boards, keratin solutions were recently shown to improve the fire resistance and, at the same time, to serve as ecological binder to greatly improve the strength [34]. Based on a reductive extraction with sodium sulfide, we report herein a way to produce keratin hydrogels by autogenous cross-linking of the extracted proteins upon drying. It was found that the drying conditions need to be carefully controlled as these significantly influence the yield and swelling properties of the obtained gels. One of these gels was tested as plant growth medium using garden cress (*Lepidium sativum*) as a model species particularly to prove the absence of toxic substances in the final gels.

## 2. Materials and Methods

### 2.1. Materials

Goose feathers were provided by Treude und Metz GmbH & Co. KG, Bad Laasphe, Germany. Feathers were washed with a commercial detergent and dried at 105 °C by the company. Feathers were cut into small pieces using a cutting mill (Retsch SM300) with a build-in 200 µm sieve before hydrolysis. Sodium sulphide (60–64% purity, analytical grade) and reverse osmosis water were obtained from VWR; hydrogen peroxide (analytical grade, 30%) was purchased by Merck. Dye-free nylon sieves with a mesh size of 90 µm from Rosin Tech Products (Bethpage, NY, USA) were used.

### 2.2. Preparation of Keratin Hydrolysate from Feathers under Reducing Conditions

To assess the rates of hydrolysis, 10 g of feather cuttings are added to 200 mL of freshly prepared Na_2_S solution in concentrations of 10, 7.5, or 5 mg/mL Na_2_S (Na_2_S/feathers = 0.2, 0.15, or 0.1 g/g), and the mixture is agitated on a shaker at 23 °C and a speed of 250 rpm. To determine the amount of dissolved keratin, samples of 3 mL each are withdrawn at regular intervals and filtered to give clear keratin solutions without undissolved feathers. A total of 1 g of this solution is dried at 70 °C in an oven to a constant weight. Besides hydrolysed keratin, the residue also contains also Na_2_S. The same was performed with a solution consisting purely of Na_2_S, yielding the dry Na_2_S weight. For pH measurements, a laboratory pH meter (Hanna Instruments Deutschland GmbH, Vöhringen, Germany) was used.

### 2.3. Preparations of Keratin Gels

Feathers are hydrolysed following the general procedure described above, but using the experimental conditions shown in Table 1. The reaction mixture is then centrifuged for 15 min at 4000 rpm, decanted, and filtered to remove all undissolved feather material. The clear solutions are dried in an oven at 70 °C for 24 h to constant weight. The resulting solid is gently crushed and added to an excess of distilled water under gentle agitation upon which the solid slowly loses its yellow colour and swells to form a keratin gel. After 10 min, the gel is separated from the solution using a nylon sieve with a mesh size of 90 µm. This washing procedure is repeated three times to remove all residual sodium sulphide. The final gel tests negative for sulphide using lead acetate paper. 

### 2.4. Swelling Experiments 

Approx. 100 mg of the dried gel is mixed with an excess of water and allowed to swell until reaching the swelling equilibrium. The swollen gels are removed by means of a nylon sieve with a mesh size of 90 µm and subsequently dried at 50 °C. From these values, the degree of swelling at equilibrium (EDS) in g/g is determined according to EDS = (m_s_ − m_d_)/m_d_, where the indices s and d denote the swollen and the dry state. The swelling/drying cycle is repeated several times to assess the change of the EDS on repeated swelling. 

### 2.5. Elemental Analysis

The elemental composition is determined on a Vario EL Cube (Elementar Analysensysteme GmbH, Langenselbold, Germany) in a helium gas stream using finely ground samples. The given values for carbon, hydrogen, and nitrogen are an average of 2 measurements of the same sample.

### 2.6. Sulphur and Carbon Analysis

Both the sulphur and the carbon content are determined using an ELEMENTRAC CS-I Carbon/Sulfur Analyzer. All samples are dried at 105 °C to a constant weight prior to the measurements. A total of 50 mg of the dry sample is mixed with iron particles and heated to >2000 °C in an induction furnace under a constant stream of oxygen. The combustion gas is analysed by infrared absorption. 

### 2.7. Amino Acid Analysis

The amino acid analysis follows the method of Spackmann, Moore and Stone [36]. For that, 20 mg of the sample are mixed with 5 mL of 6 N hydrochloric acid and 0.1% aqueous phenol solution, which serves as an antioxidant. The mixture is heated for 24 h at 110 °C. After cooling, the hydrolysate is transferred quantitatively into a 10 mL volumetric flask. The excess of hydrochloric acid is removed on a rotatory evaporator at 50 °C and the hydrolysate is washed several times with distilled water. The solution is evaporated to dryness, the flask purged with nitrogen, and the residue dissolved in a lithium acetate buffer at a pH of 2.2. Afterwards, amino acid analysis is performed by ion exchange chromatography after column derivatisation using ninhydrin. Due to acidic hydrolysis, glutamine and asparagine are present as glutamic and aspartic acid and are measured together. Tryptophan is destroyed due to acidic hydrolysis.

### 2.8. SDS-PAGE Analysis of Extracted Keratin

Electrophoresis was performed on 4–20% Mini-PROTEAN TGX precast gels (Bio-Rad Laboratories, Inc., Hercules, CA, USA). Samples were prepared with 2× Laemmli sample buffer (Bio-Rad Laboratories, Inc.). A broad-range unstained SDS-PAGE standard with a mixture of 12 recombinant proteins (2–250 kD) (Bio-Rad Laboratories, Inc.) was used as a reference in a separate well. Gels were run using the Mini-PROTEAN Tetra electrophoresis cell at 200 V until the dye front reached the bottom of the gel. Both types of gels were run using Tris/Glycine/SDS running buffer [25 mM Tris, 192 mM glycine, 0.1% (*w*/*v*) SDS] (Bio-Rad Laboratories, Inc.). Gels were stained with Bio-Safe™ Coomassie stain (Bio-Rad Laboratories, Inc.). 

### 2.9. Infrared Spectroscopy (FTIR-ATR)

IR spectra were recorded using a Bruker Alpha-P FTIR spectrometer equipped with a diamond ATR window (BrukerOptik GmbH, Ettlingen, Germany). All spectra were recorded in the spectral range of 4000–400 cm^−1^ with 16 scans at a spectral resolution of 2 cm^−1^ resolution. Before each measurement, the diamond ATR crystal was cleaned with isopropanol.

## 3. Results and Discussion

### 3.1. Feather Keratin Hydrolysis with Sodium Sulphide

**Figure 1:**
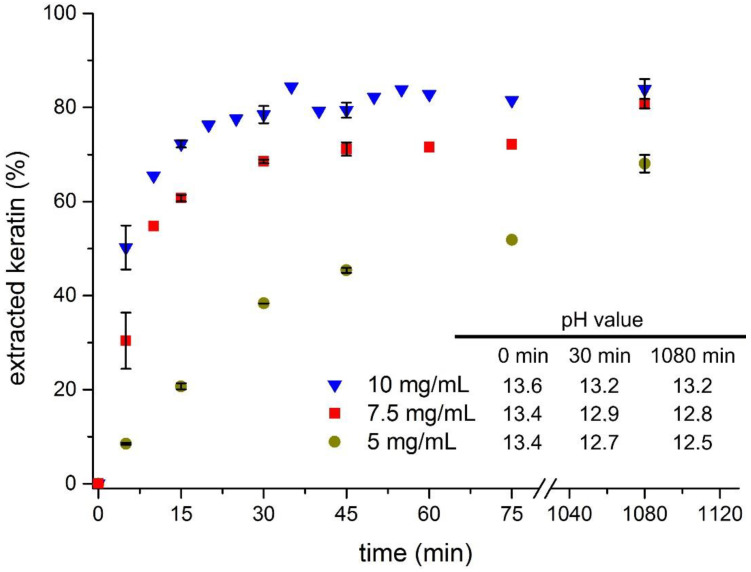
Progress of the feather dissolution as a function of time and Na_2_S concentration at 23 °C. The inset shows the corresponding pH values at three points of time during the reaction.

To find suitable conditions for the dissolution of feathers under reducing conditions, the amount of solubilised protein was determined as function of time and reagent concentration (Figure 1). For economic reasons, Na_2_S was chosen to cleave the disulphide bonds in keratin rather than, e.g., the expensive 2-mercaptoethanol. Prior to the hydrolysis, the crude feathers were cut to a size of 200 µm to facilitate the mixing of feathers and reagent solution. In a previous study, a concentration of 10 mg/mL was found to enable a fast hydrolysis with high yields and, therefore, this concentration was used as a starting point [29]. The feather-to-solution ratio was increased to 1:20 to assure homogenous wetting of the feathers directly after mixing. This calculates to a Na_2_S/feathers weight ratio of 0.2 g/g or Na_2_S/protein sulphur ≈ 3.6 mol/mol. Under these conditions, the reaction starts immediately, as indicated by a noticeable decrease of the dispersion viscosity. The amount of dissolved keratin increases rather fast, reaching 50% after 5 min and 79% after 30 min. Beyond that, the amount of dissolved keratin increases only slowly and finally levels off at approx. 84%. Still, this is a noticeable increase in dissolved keratin compared to the mentioned reference. However, as 10 mg/mL is a rather large excess it was of interest to study the progress of the hydrolysis at lower amounts of Na_2_S. Here, the reactions slow down and suffer from reduced yields, although the effect is not yet pronounced with 7.5 mg/mL (0.15 g/g, 2.7 mol/mol). After 30 min, the amount of dissolved keratin is 70% and reaches 80% after 18 h. Using 5 mg/mL (0.1 g/g, 1.8 mol/mol), the curve reaches only 39% after 30 min and 68% after 1080 min. Since the pH value of the solution was previously found to be a key parameter for a fast keratin hydrolysis, it was checked at three points of time during the present hydrolysis reactions (inset in Figure 1) [7]. Due to the low p*K*_B_ value of S^2−^, all solutions start at rather high pH values (>13) and, as expected, the values decrease with decreasing concentration and increasing reaction time. The difference between *t* = 0 min and *t* = 1080 min also increases with decreasing concentration and reaches almost 1 unit at 5 mg/mL. These observations correlate with the observed hydrolysis rates, particularly in the initial stage, and underline the previous finding that the pH value is a major factor influencing the keratin dissolution.

During the work on this graph, it was observed that some of the dried hydrolysate samples formed a rather dense film and some dissolved when treated with water. While this was expected, some samples formed hydrogels by themselves, which was quite surprising and is, to the best of our knowledge, not reported in the literature so far. As the concentration of 10 mg/mL enabled a fast and reliable feather hydrolysis that was mostly finished after 30 min, it was used as base case for further experiments.

### 3.2. Keratin Hydrogel Formation by Drying

**Figure 2:**
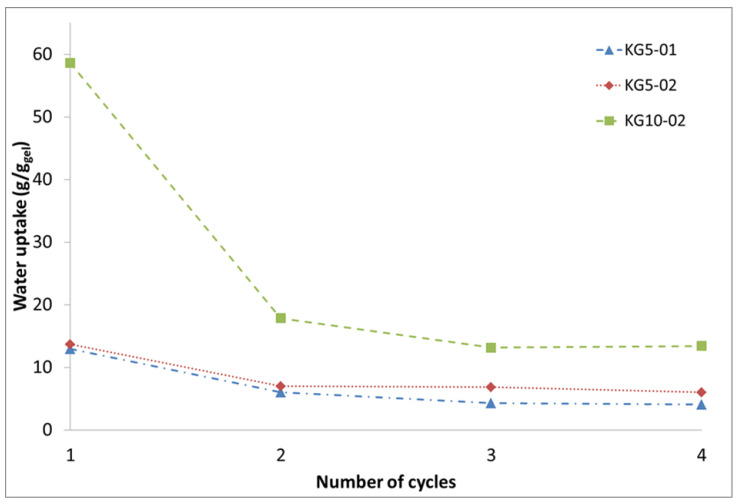
Trend in water uptake of keratin hydrogels in relation to their weight obtained after drying.

Based on this unprecedented finding, four conditions were used to assess the formation of keratin hydrogels and their properties. The samples KG5-01, KG5-02, and KG5-04 are used to study the influence of the Na_2_S to keratin ratio, while KG10-02 is similar to KG5-02 albeit double-feather concentration. The exact compositions are given in Table 1. The reaction time was fixed to 30 min as this promised a high yield of soluble protein at reasonable level of backbone hydrolysis. Increasing the Na_2_S/feather ratio from 0.1 g/g (KG5-01) to 0.4 (KG5-04) primarily increases the yellow colouration of the solid residues obtained after drying. This is an effect of the increased amount of unreacted Na_2_S in the product. At the same time, the amount of leftover material is reduced, which is the result of an increasing extent of hydrolysis. Thus, while the dry residue of KG5-01 and KG5-02 forms a cracked film covering the bottom of the vessel, the residue of KG5-04 is only found around the edges of the vessel (Figure S1 in the Supplementary Material). The appearance of the residue received from KG10-02 is somewhere between those of KG5-02 and KG5-04, despite having the same Na_2_S to feather ratio as KG5-02. The difference is most likely the result of the reduced water to feather ratio, which could decrease the efficacy of the hydrolysis yield and lead to an increased amount of Na_2_S in the dried residue. After submerging the solids in water, the yellow discolouration disappears as the water-soluble Na_2_S dissolves, and some of the protein material is obtained as a gel. The question thus arises as to how the cross-linking reaction to form a gel takes place. Previously, the cross-linking was assigned to the oxidation of the free thiolate/persulphide anions on the protein chains by molecular oxygen upon drying. However, if the dry residues are dissolved in a dilute hydrogen peroxide solution, the yield of gel increases significantly at the cost of the swelling degree. This suggests that cross-linking is at least partially also possible after the drying step. In terms of the degree of swelling, KG5-01 and KG5-02 are similar, being able to take up approx. 13 times their weight in water (Figure 2). Qualitatively, KG5-02 produces larger amounts of gel than KG5-01. The reason is the amount of dissolved keratin, which increases with increasing Na_2_S to feather ratio (cf. Figure 1). However, when this ratio is further increased (KG5-04), no hydrogel is obtained after submerging the solid in water. Although no kinetic analysis was made for this high ratio, it can safely be assumed that the hydrolysis is so fast that large amounts of small protein fragments are produced even early in the reaction. These are not able to cross-link into 3D networks upon drying. When the amount of feathers is doubled, as in KG10-02, the solid residue transforms into large amounts of gel, which take up 60 times their weight in water (Figure 2).

However, repeated swelling and drying shows a significant decrease in water absorption (Figure 2). The effect seems to be dependent on the original degree of swelling of the gels and levels off after 2 to 3 cycles. For KG5-01 and KG5-02, the final degree of swelling is approx. half the initial one (5–8 g/g). However, for KG10-02, a massive decrease is observed, dropping from 60 to 17 g/g in the third cycle. The decrease could be due to reorganisation processes in the keratin gel, e.g., to form larger fractions of crystalline beta sheet structures, which increases the apparent degree of cross-linking and thus lowers the swelling ratio. Alternatively, the extended contact with atmospheric oxygen could increase the amount of disulphide bridges with a similar effect. The decrease is not due to a continued loss of soluble protein material or degradation as the dry weight decreases only marginally with increasing number of swelling cycles.

**Figure 3:**
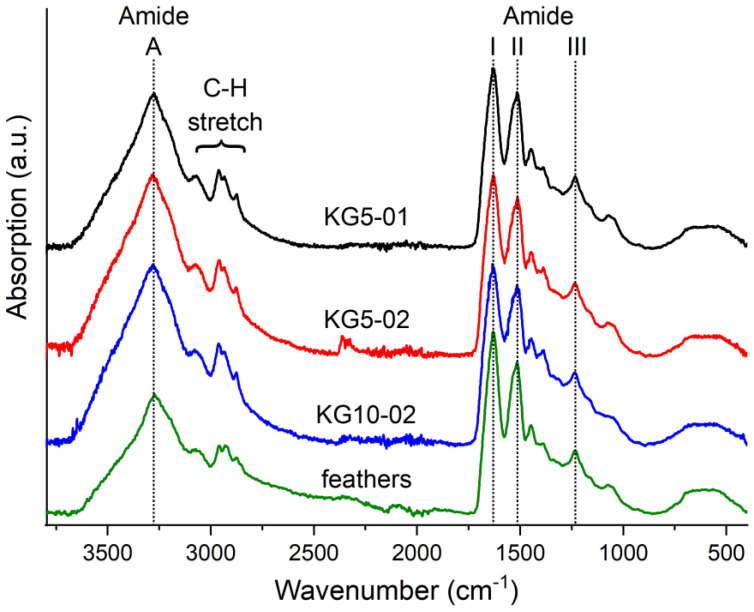
ATR-FTIR spectra of dry keratin hydrogels and raw feathers.

To get more insight into the gel structure, the secondary structure of the protein was analysed by ATR-FTIR (Figure 3). All samples show the typical protein vibrations such as the prominent N−H stretching vibration (amide A) at 3275 cm^−1^, the C=O stretching vibration (amide I) in the region of 1630 cm^−1^, the amide II (C−N stretching) at 1515 cm^−1^, and amide III (N−H bending) at 1230 cm^−1^ [37].

Among these, the position of the amide I band is highly influenced by the secondary structure of the protein. In the untreated feathers, the maximum is found at 1630 cm^−1^. This is in accordance with the β-sheet secondary structures that are found in the range of 1623 to 1641 cm^−1^ in ^1^H_2_O [38]. Deconvolution of the amide I band could be used to further differentiate the secondary structure. However, due to the low resolution of the ATR spectra of 4 cm^−1^, we decided against this. For all dried gels, the amide I band appears at 1630 ± 2 cm^−1^. It is free of distinct shoulders and the position of the IR bands is found to be unaffected by the Na_2_S to feather ratio. This leads to the conclusion that the structure of the dry gels is governed by β-sheets [39]. As shown by Popescu et al., this is not uncommon even for smaller keratin fragments that can show crystal structures similar to the original feathers [40]. It is assumed that the β-sheets formed during drying are preserved during swelling. This would corroborate the observed reduction of the degree of swelling (Figure 2). The IR spectra show no signs of the cysteine S−H stretching band at 2540 cm^−1^ indicating the absence of free cysteine in the gels [41]. Although the S–S stretching vibration of cystine is not IR active [42] and can, therefore, not directly be observed, it can be safely assumed that the disulphide bridges have reformed during drying. This is also corroborated by the amino acid analysis shown in the Supplementary Material.

### 3.3. Influence of Drying Conditions 

When trying to prepare larger amounts of gel, a rather peculiar observation was made. The evaporation of 20 times the volume of KG10-02 solution (compared to the experiments in the previous chapter) under otherwise identical conditions did not yield any hydrogel. Similarly, the evaporation of 20 times the volume of KG5-02 solution produced a clear hydrogel with an increased degree of swelling. The causes of this appeared to be the rate and the conditions of evaporation. To better understand this process, solutions of KG5-02 were dried with different volume to surface ratios, as this affects the evaporation time.

**Figure 4:**
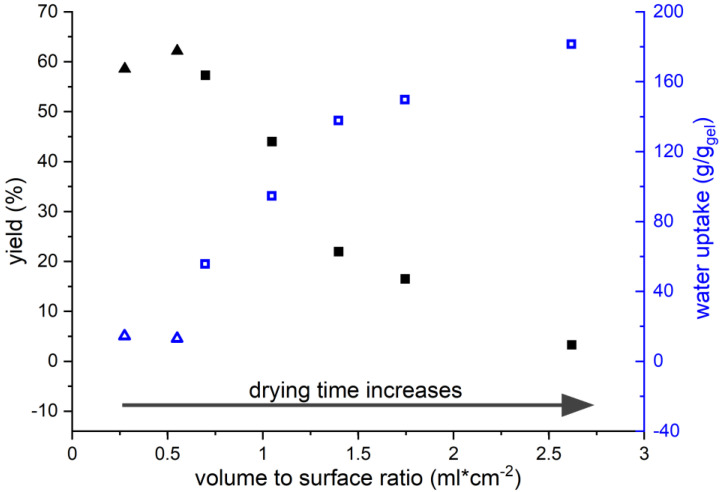
Trends in gel yield (solid) and water uptake (open) using different volume to surface ratios for the drying of KG5-02 solutions. Triangles refer to gels prepared in a wide, shallow petri dish (d × h = 136 mm × 20 mm) and squares to gels prepared in narrow, high cylindrical vial (d × h = 27 mm × 430 mm).

**Figure 5:**
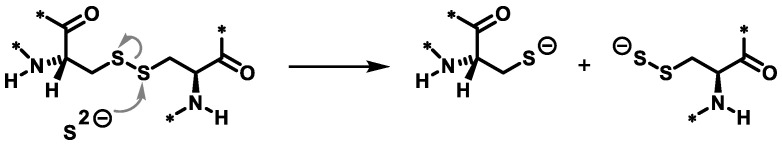
Reaction scheme for the disulphide bond cleavage by a S^2−^ anion. The stars denote the continuation of the protein chain.

Initial experiments were made using a petri dish with a diameter of 136 mm and an edge height of 20 mm (Figure 4, triangles). The wide, shallow geometry favours an unhindered and fast evaporation. This gives rise to high yields of gel with only low water swelling capacities. Due to the geometry of the petri dish, a higher volume to surface (vol/sa) ratio was not possible. The next experiments were, therefore, carried out in a narrow, high cylindrical vial with a diameter of 27 mm and a height of 430 mm. Previous studies by Stefan on evaporation indicate that, if the distance of the liquids surface to the rim is greater than the diameter of the vessel, then the evaporation rate slows down and is then governed by molecular diffusion [43]. This is because the air stream can no longer transport the air saturated with water out of the vessel through convection. Thus, evaporation from our cylindrical vial proceeds at a much slower rate than from the petri dish. The consequences are particularly apparent when comparing the gels made at vol/sa = 0.55 (petri dish) and at vol/sa = 0.7 (vial). Despite very similar starting conditions and yields, the gel prepared in the vial shows an approx. three times higher degree of swelling. With increasing volume to surface ratio, the degrees of swelling continuously increase up to 180 g/g. However, at the same time, the yield decreases significantly down to only 3%. This means, only a very small part of the keratin is still able to form a cross-linked network and the rest dissolves during the washing process. Both effects can be explained by the reduced evaporation rate in the vial, which increases the contact time between the protein and the reaction solution. Generally, the solution containing Na_2_S acts in two ways on the protein: the S^2−^ anion attacks the disulphide bridge and cleaves it according to Figure 5 by forming a thiolate and a persulphide anion [44].

**Figure 6:**
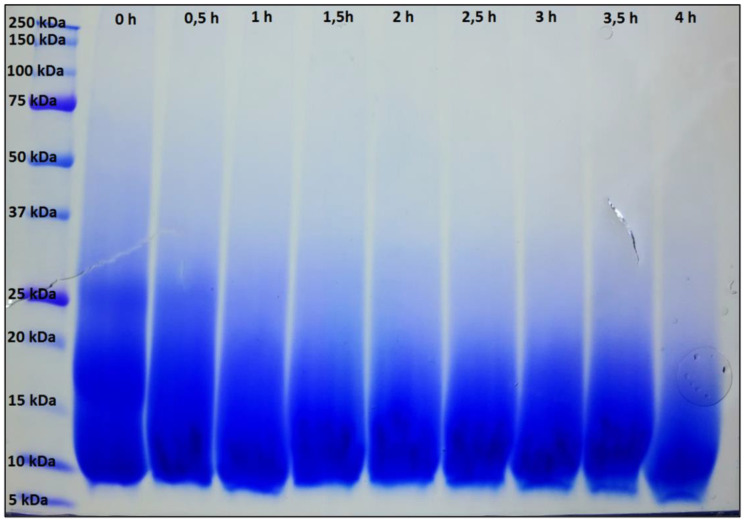
SDS-PAGE pattern of keratin solution with sodium sulphide at different times in the drying process at 70 °C. The left column shows the molecular weight standard. The lines next to the standard show the molecular weight of the standard for 0 to 4 h of the drying process.

In addition, the solution is alkaline due to the protolysis equilibrium S^2−^ + H_2_O → HS^−^ + OH^−^ and hydroxide is known to cleave the amide bonds of the backbone. Although this process is slower than the nucleophilic attack of S^2−^, the pH of the solution rises as the solution gradually evaporates. However, the rate of the nucleophilic attack also increases with increasing pH [45]. The formation of persistent gels requires the polymer chains to be cross-linked three-dimensionally. Generally, higher degrees of cross-linking give rise to materials with lower degrees of swelling and vice versa, but below a critical point no persistent gels are formed. It is important to keep in mind that in the present system cross-linking is not limited to the disulphide bridges but can also be provided by crystalline domains, e.g., in the form of β-sheets. These constitute an estimated amount of approx. 30% of the fowl protein [35] and their presence in the gels was confirmed by IR spectroscopy (Figure 3). The β-sheet forming domains are mainly located between amino acids 31 and 90 (Figure S2 in the Supplementary Material). To better understand the correlation between contact time and yield as well as the degree of swelling, the evolution of the molecular weight distribution of the protein was analysed during drying at 70 °C (Figure 6).

The original KG5-02 hydrolysate (*t* = 0 h) showed a broad molecular weight distribution ranging from 10 kDa to 30 kDa. The literature reports the molecular weight of goose feather keratin to be approx. 10 kDa and thus higher molecular weights represent dimers, trimers, or higher agglomerates [46]. After 0.5 h at 70 °C, the bands at 25 kDa and above begin to fade. After 1 h, the trimers appear to be mostly gone. After that, not much change in the appearance of the pattern is observed up to 2.5 h. This initial period seems, therefore, to be governed by a breakdown of the agglomerates rather than hydrolysis of the protein backbone. Beginning at 2.5 h, the bottom edge of the distribution starts to drift towards lower molecular weights. The effect is clearly visible at 4 h and indicates alkaline hydrolysis of the protein backbone. At the same time, the bands at higher molecular weights seem to disappear so that after 4 h, only monomers and some dimers are present in solution alongside larger amounts of smaller fragments. In the goose-feather protein, 6 of the 7 cysteine units are located among the first 26 amino acids, while the 7th forms the C-terminus (Figure S2 in the Supplementary Material) [35]. Random scission of protein chains initially gives, with high probability, fragments with more than one thiol group and soluble pieces. The former can form gels and the latter are lost to the aqueous phase. At the same time, chain scission in the centre and/or towards the C-terminus reduces the ability to form β-sheets that contribute to the cross-linking. Increasing contact time with the alkaline Na_2_S-containing solution increases the extent of hydrolysis. That is, the sequences able to form β-sheets become shorter and the number of fragments with multiple thiol groups decreases. This leads, on the one hand, to an increase in soluble fragments, which explains the decreasing yield of gel in Figure 6. On the other hand, it leads to a reduction in the cross-linking density. The latter clarifies the increasing degree of swelling up to a critical point where no more persistent gels are formed and almost all of the protein material dissolves. It should be noted that the trend of the swelling behaviour matches in principle that reported by Poole, who found his protein films becoming sturdier when only short solubilisation times (0.5 to 1 h) were used [29]. Poole and co-workers commented that alkaline degradation of the backbone should be prevented for mechanically stable materials. Conversely, we here use extended hydrolysis to obtain hydrogels.

Although β-sheet formation can contribute to the cross-linking, the thiol groups of cysteine and their ability to form disulphide bridges under mild oxidation conditions during drying will certainly have a greater impact. To get more insight into potential changes in the sulphur content as a result of the hydrolysis and the drying, the elemental composition of the feathers, the KG5-02 hydrolysate, and a highly swelling gel (150 g/g) were analysed. In order not to falsify the results by the excess Na_2_S, the hydrolysate was dialysed before the analysis. The feathers used in this study were found to have a total cysteine content of 9.9%, which is slightly lower than other values for goose feathers stated in literature, but still higher than the cysteine content reported for, e.g., chicken feathers [47]. Table 2 and Table S1 in the Supplementary Material show, that the C, H, and N content stays rather constant. However, the sulphur content increases in the order feathers < hydrolysate < gel to more than twice the original value. The elevated amounts of sulphur match an observation by Goddard under similar conditions, but he could rule out the presence of elemental sulphur [7]. The present gels were tested with lead acetate and were found not to contain any residual sodium sulphide. A number of effects are plausible for this. One is the selective loss of amino acids other than cysteine during the process, which increases the relative cysteine content and with it the sulphur content in the residue. While this cannot be completely ruled out, it is unlikely particularly for the hydrogel. An increased cysteine content entails an increased cross-linking, which in turn decreases the degree of swelling. This is not observed. Moreover, the amino acid analysis (Table 2, Column 5, and Table S2 in the Supplementary Material) indicates a slightly decreased cysteine content in the hydrolysate and a significant decrease in the gel. The higher sulphur content in the hydrolysate and in the gel is, therefore, more likely explained by the formation of persulphide anions following a nucleophilic attack of S^2−^ on the disulphide bridge as outlined in Figure 5 [45]. A consequence of these persulphides is that the oxidation during gel formation can give rise to tri- and, much less likely, higher oligosulphide bridges between the protein chains [48]. These are longer than the original disulphide bridges and, thus, create a wider-meshed network. This might also contribute to the observed increase in the degree of swelling. Another interesting result of the amino acid analysis is that the amount of cysteic acid triples during gel formation (Table S2 in the Supplementary Material). The occurrence of cysteic acid is most likely the result of the extended exposure of the reaction solution to atmospheric oxygen during drying. Admittedly, the overall amount of cysteic acid is rather small so that this being an artefact cannot be completely ruled out. However, if the value is correct, this would support the previous claim that gel formation is, at least, partially caused by oxidation of thiol/persulfide groups. In addition, the presence of sulphonate groups in the gel structure would also contribute to the observed increase in the degree of swelling.

The loss of cysteine is most likely due to β-elimination forming dehydroalanine [49]. However, lysinoalanine, a common derivative of dehydroalanine that can contribute to protein cross-linking, cannot form due to the absence of lysine in feather keratin. Lanthionine, on the other hand, the product of a thiol-ene reaction between cysteine and dehydroalanine could form and would then contribute to cross-linking as well as the reduction in the cysteine content [50]. Unfortunately, the analyses carried out on the gel are not able to detect lanthionine. As well as cysteine, the amino acid analysis (Table S2 in the Supplementary Material) detected major changes in the threonine (−38%) and serine (−42%), as well as the alanine (+33%) and glycine (+26%) content when comparing feathers and gel. Of these, the evolution of the serine content is most notable, since it first increases during hydrolysis and then decreases strongly. The slight increase could be the result of the transformation cysteine → serine, while the strong decrease could originate in an E1cb-type elimination to form dehydroalanine upon increasing pH value during evaporation [51]. Threonine and serine both being hydrophilic amino acids could, on the other hand, also be lost as water soluble fragments during washing as only the gel-bound protein was subject to amino acid analysis. The reason for the increase in alanine and glycine is not entirely clear, but since both are less polar amino acids, a reasoning opposite to the one just given for the loss of serine and threonine could be envisioned. 

### 3.4. Prove-of-Principle Experiment

**Figure 7:**
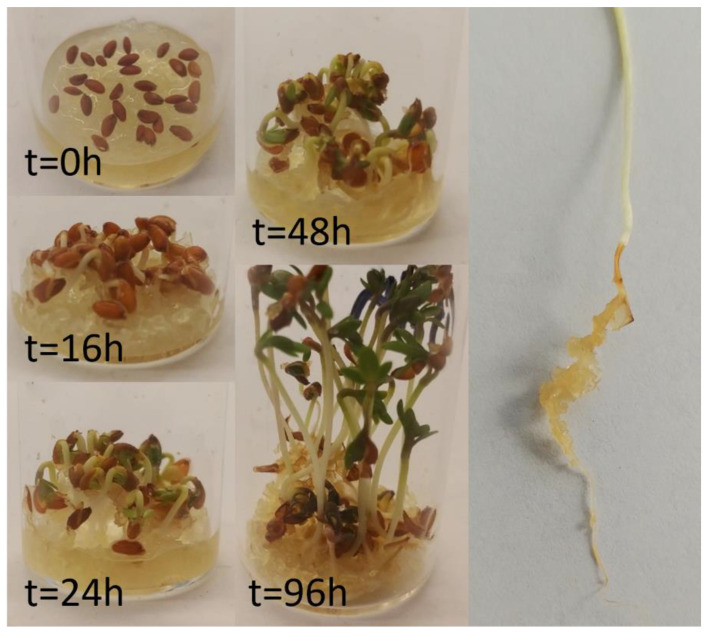
Growing of cress on keratin hydrogel at different time intervals. The picture on the right shows the cress-root which is grown on the keratin gel particles.

With the keratin gels in hand, a potential application as plant growth media was studied using cress, not only because it is a fast-growing plant, but also because it is used as an indicator for pollutants in the so-called cress germination test (Figure 7) [52]. Thus, if traces of harmful substances such as Na_2_S were left in the gels, cress would not grow properly. For the gel, a KG10-02 solution was dried at vol/sa = 1. However, when upscaling the conditions from Figure 4, it must be taken into account that the evaporation rate is proportional to the surface to the power of 0.9 [53]. That is, when doubling the surface, the rate of evaporation increases only by a factor of 1.87 despite the same surface to volume ratio. This is particularly important on a larger scale. For the proof of principle experiment, the seeds were simply placed on the swollen gel. Commercial cress is reported to have a germination probability of 90%. After 24 h, 27 out of 30 seeds had started to grow. This matches the reported probability and indicates the absence of harmful substances in the gels. The cress grew fast and reached its full height after 96 h. During this time, the hydrogel acted as a water reservoir and consequently, a slow shrinking of the gel was observed starting after approx. 16 h. To compensate this and potential evaporation, 0.2 mL of water were added every 24 h. However, the drying of the gel was found to have no negative effect on the plant growth, since the roots stay wet until no more water is available in the hydrogel. As shown in Figure 7, the plant roots grow into the hydrogel, which firmly adheres to the full length of the roots. This enables better water utilisation by the plant.

## 4. Conclusions

The use of sodium sulphide (Na_2_S) to solubilise the keratin is crucial as this preserves the cysteine thiol groups and affords a protein solution that can cross-link autogenously when exposed to air. A purification step of these solutions, e.g., by dialysis, is not required. This makes the process not only fast, but also cost effective. Controlling the total contact time (tct) of keratin and reaction solution is critical as this determines the product properties and yields. In the present case, the total contact time consists of the hydrolysis itself and the subsequent drying. Owing to our experimental setup, the latter was more influential. Generally, longer tct affords less material with a higher degree of swelling although this can partially be counteracted by using hydrogen peroxide during gel formation. Initial tests showed the gel to be suitable as plant growth media. The plant roots grow into the gel, which encloses them completely and adheres to them firmly. The gels also serves as water reservoir in dry periods. It is thus not only a value-added product made from waste, but even more a fabricated plant growth medium as well as a potential additive to improve inferior soils.

## Figures and Tables

**Table 1:** Experimental conditions for the preparation of keratin hydrolysate as source for keratin gels.

Sample Name	Feathers ^a^	Na_2_S	Na_2_S/Feathers	
	g	g	g/g	mol/mol ^b^
KG5-01	5	0.5	0.1	1.8
KG5-02	5	1	0.2	3.6
KG5-04	5	2	0.4	7.1
KG10-02	10	2	0.2	3.6

^a^ Sample volume 100 mL, shaking time 30 min; ^b^ calculation based on 7 cysteine units in the protein chain and a molecular weight of 9756.08 g/mol [35].

**Table 2:** Carbon and sulphur content analysis for feathers and keratin hydrogels.

Sample Name	C [wt%]	S [wt%]	S/C Ratio (×10^−2^)	Total Cysteine ^a^ mol/100 mol
feathers	46.0	1.47	3.2	9.9
KG5-02 dialysed	46.7	2.68	5.7	9.4
Keratin hydrogel water uptake 150 g/g	47.7	3.26	6.8	7.8

^a^ See Table S2 in the Supplementary Material for full amino acid analysis.

## Data Availability

Data is available from the authors upon request.

## Supplementary Materials

The following are available online at https://www.mdpi.com/article/10.3390/polym13203581/s1, Figure S1: Solids formed after drying at 70 °C of keratin solutions from sodium sulphide extraction and keratin gels yielded by them after washing with water in swollen state. Figure S2: Amino acid sequence of fowl keratin according to [35]. The marking of β-sheet stabilizers and destabilizers was made according to Koehl, P.; Levitt, M.; *Proc. Natl. Acad. Sci. USA* **1999**, *26*, 12524–12529. Table S1: Elemental analysis of feathers and keratin hydrogels. Table S2: Amino acid analysis of raw feathers, extracted keratin (cleaned through dialysis) and keratin hydrogel with high water uptake.
