# Supplementary material for: Autogenous Cross-Linking of Recycled Keratin from Poultry-Feather Waste to Hydrogels for Plant-Growth Media
Source: Polymers (Basel). 2021 Oct 17;13(20):3581. doi: 10.3390/polym13203581 (PMC8540439; doi:10.3390/polym13203581)
Supplement: Supplementary file 1 [file polymers-13-03581-s001.zip › polymers-1381289-supplementary.pdf]

Supporting Information

To

Autogenous cross-linking of recycled keratin from poultry-feather waste to hydrogels for plant-growth media

Markus Brenner and Oliver Weichold

Institute of Building Materials Research
RWTH Aachen University
Schinkelstraße 3, D-52062 Aachen, Germany

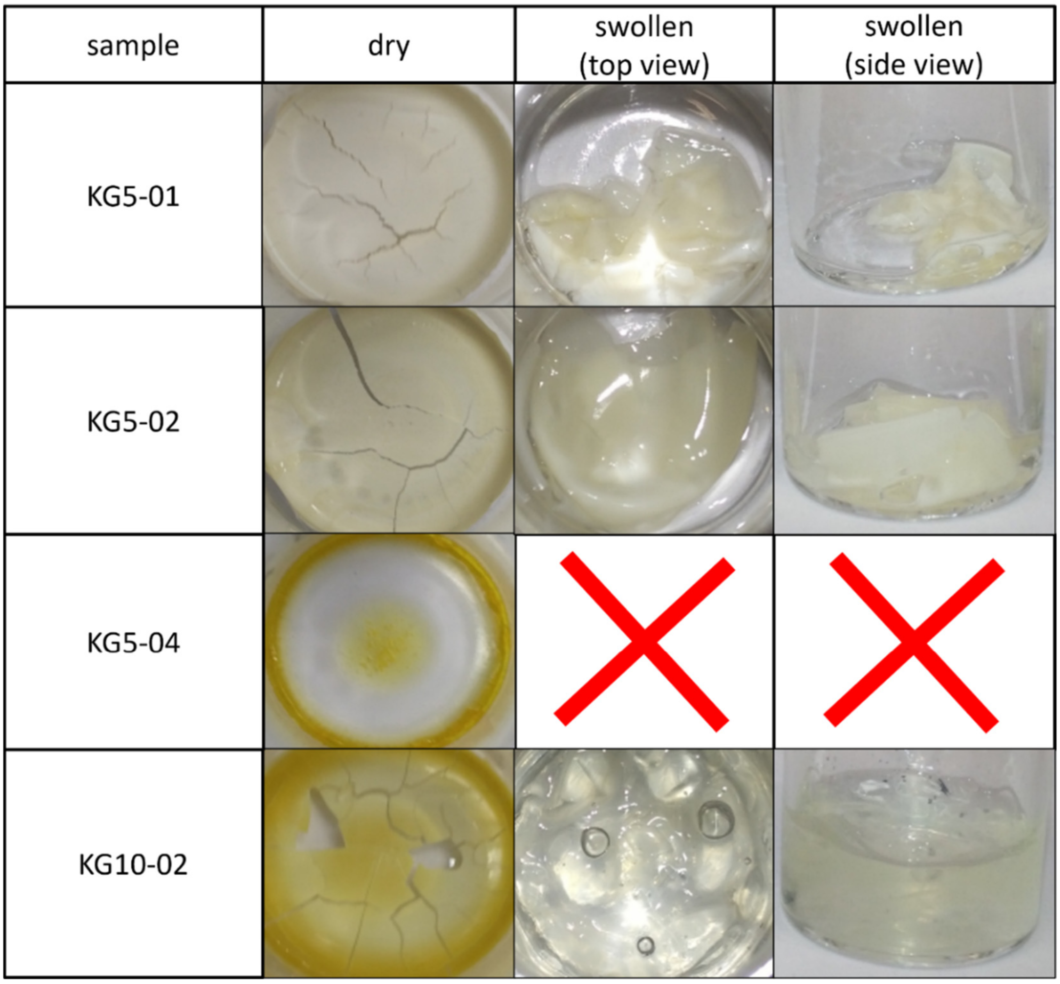

Figure S1. Solids formed after drying at 70 °C of keratin solutions from sodium sulfide extraction and keratin gels yielded by them after washing with water in swollen state.

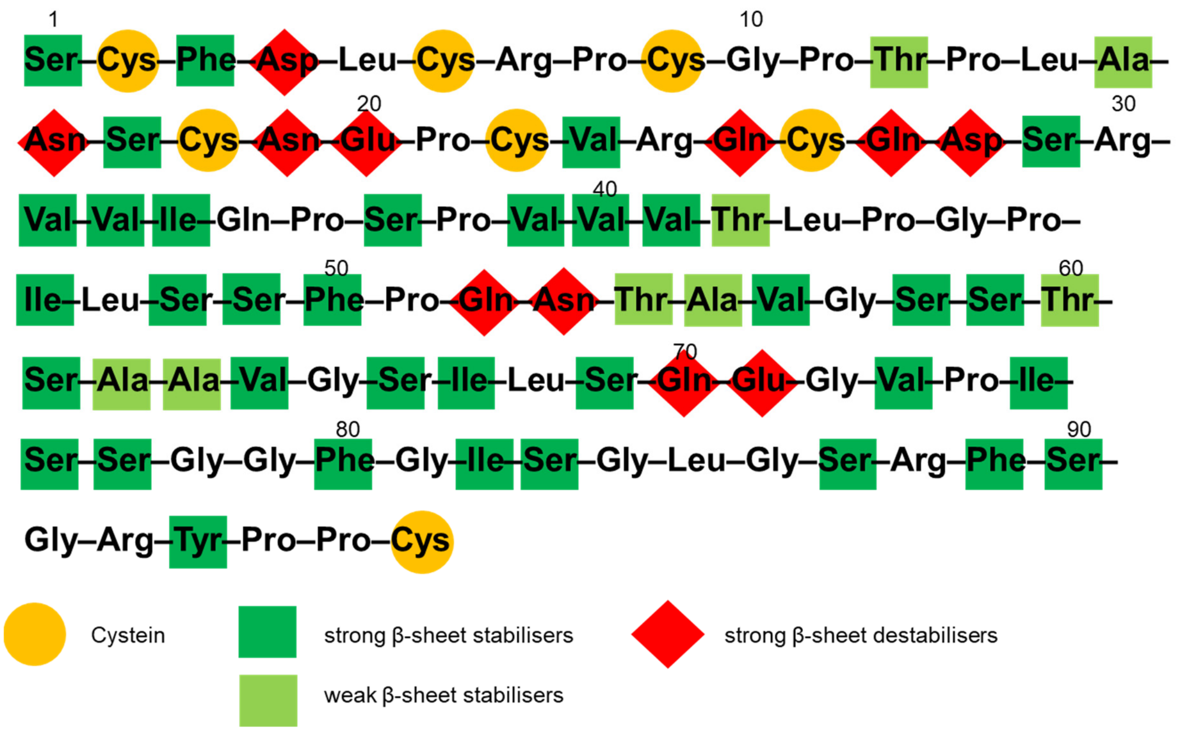

Figure S2. Amino acid sequence of fowl keratin according to K. M. Arai, R. Takahashi, Y. Yokote, K. Akahane, *Eur. J Biochem.* **1983**, *132*, 501–507. The marking of β-sheet stabilizers and destabilizers was made according to P. Koehl, M. Levitt, *Proc. Natl. Acad. Sci. USA* **1999**, *26*, 12524–12529.

Table S1. Elemental analysis of feathers and keratin hydrogels.

Name	C [wt%)	H [wt%]	N [wt%]
Feathers	47.62	6,73	15,15
Keratin (dialysed)	46,50	6,70	14,98
Keratin hydrogel<br>water uptake 150 g/g	47,66	6,99	15,12

Table S2. Amino acid analysis of raw feathers, extracted keratin (cleaned trough dialysis) and keratin hydrogel with high water-uptake.

	**Amino acid analysis (mol/100 mol)** [c]		
	Feathers	KG5-02[a]	Keratin hydrogel water uptake of 150 g/g
$CysO_3^-$	0,1	0,1	0,3
$(1/2Cys)_2$	9,8	9,3	7,5
Cys	0,0	0,0	0,0
**Cys (total)**	**9,9**	**9,4**	**7,8**
Asp [b]	6,6	6,4	7,2
Thr	5,0	5,0	3,1
Ser	13,7	14,7	7,9
Glu [b]	6,8	6,5	7,2
Pro	11,9	12,2	13,8
Gly	13,3	13,5	16,8
Ala	5,8	5,5	7,7
Val	8,1	8,0	8,0
Met	0,1	0,1	0,0
Ile	4,4	4,3	4,4
Leu	7,6	7,6	8,3
Tyr	3,2	3,5	3,3
Phe	3,1	3,1	2,9
Orn	0,2	0,1	0,4
Lys-Ala	0,0	0,0	0,0
Lys	0,7	0,2	0,1
His	0,3	0,2	0,2
Arg	4,2	4,4	4,5
**Total**	**100,0**	**100,0**	**100,0**

[a] Determined on dialysed solution. [b] Due to the acidic conditions during the analysis, glutamine and asparagine hydrolyse to glutamic and aspartic acid and cannot be determined individually. [c] Tryptophan is destroyed during the acidic hydrolysis.
